# Practical Notes on Diagnosis and Treatment

**Published:** 1907-01-05

**Authors:** 


### PRACTICAL NOTES ON DIAGNOSIS AND TREATMENT.

**Idiosyncrasy to Potassium Iodide.**

WITH patients who have a specific idiosyncrasy against potassium iodide this may often be minimised by combining the drug with aromatic spirits of ammonia and a quinine preparation, and by administering it in considerable quantities of water. *Sir Felix Semon.*

**Pustular Eczema of the Hands.**

IN this condition I get the best results with ammoniated mercury ointment (half the official strength) as a local application and carbonate of iron internally.—*Mr. Shield.*

**Picrotoxine in Night-Sweats.**

PICROTOXINE is useful in the night sweats of phthisis and more particularly in the later stages of the disease. It is then often more efficacious than atropine. It may be given in granules each containing $\frac{1}{100}$ grain. Two to four of these may be given daily and the treatment continued for several days.

**Capillary Pulsation.**

THIS may be well observed through a glass slide pressed gently against the lip. It is a characteristic incident in the course of aortic regugitation, but it is also often well seen in anæmia and in convalescence from acute febrile disorders.—*Dr. J. S. Bristowe.*

**Pyo-Salpinx without Pyrexia.**

YOU may have a considerable pyo-salpinx with a normal temperature. I have seen a case in which one tube contained 4 oz. and the other 5 oz. of pus, and yet the temperature was normal. On the other hand, with a very limited amount of purulent fluid there may be a decidedly hectic temperature.—*Dr. Handfield-Jones.*

**Hæmoptysis in Phthisis.**

HÆMORRHAGE occurring in phthisis is very liable to be followed by an increase of the tuberculous disease either in the same or in other districts of the lung. This is probably due to fresh infection by means of the inhaled blood, containing as it does the tubercle bacilli.—*Dr. Green.*

**Pneumonia in Childhood.**

PNEUMONIA in children is sometimes a very obscure disease, as the physical signs are apt to be delayed, incomplete, or uncertain. The child is flushed, with hot, dry skin, rapid pulse and respiration, and a high temperature. Even so late as the second or third day there are frequently no distinctive physical signs. Sometimes as early as the fourth or fifth day, without any physical signs having appeared, the fever terminates by crisis, and you thus get the assurance that the disease is lobar pneumonia.—*Dr. Sturgess.*

**Sciatica.**

A CASE is reported in which complete relief followed the use of nitroglycerine after many other remedies had proved of no avail. The drug was given in a 1 per cent. alcoholic solution, of which one, gradually increased to 5 minims were given thrice daily.

**Appropriate Vehicles for Individual Drugs.**

CERTAIN drugs always give better results when administered in certain vehicles. My experience has led me to believe that potassium iodide acts most effectually when given in the compound decoction of sarsaparilla, and a small quantity of quinine, when presented in the form of Warburg's tincture, has a far greater effect than when given in the solid form, or in an ordinary solution. —*Mr. Malcolm Morris.*

**Leeching in Cyanosis.**

WITH marked distress of breathing and increasing cyanosis, such as are apt to develop in children when attacked by bronchitis, leeching is of great value. At least five or six leeches should be applied.

**Empyema.**

IN empyema I think mere aspiration is waste of time, and that, as soon as we have found by the exploring needle that pus exists, a complete opening should be made to give a channel for the escape of the pus, and for the prevention of reaccumulation.—*Dr. Gilbart Smith.*

**Alopecia Areata.**

As a rule cases of alopecia areata end in recovery and treatment is not a matter of much consequence except to encourage the patient. I, as a rule, order oil of turpentine to be rubbed into the scalp, and this acts as a rubefacient. Of course, if the patient's health is bad, this must receive attention. Some cases do not recover until after many years.—*Dr. Cavafy.*

**The Knee-Jerk in Functional Disease.**

IN neurasthenia and hysteria there is apt to be considerable exaggeration of the knee-jerk. On the other hand, it should never be concluded that hysteria is a satisfactory explanation of an absent knee-jerk.

**Treatment of Irritable Ulcer.**

ABOUT the inner ankle particularly we are apt to have a small irritable ulcer which is excruciatingly painful, keeps the patient awake at night, and leads to great irritation. If on examining the ulcer with a probe there is one point at which the patient cries out, the best thing is to snip away the granulation at this point with a pair of scissors. Otherwise nitrate of silver solution (2 grains to the ounce), applied on lint and covered with a wet dressing, is the best application. But in addition opium must be given internally.—*Mr. Christopher Heath.*

